# Accumulation of natural killer cells after hepatic artery embolisation in the midgut carcinoid syndrome.

**DOI:** 10.1038/bjc.1995.120

**Published:** 1995-03

**Authors:** B. Wängberg, H. Ahlman, U. Tylén, O. Nilsson, S. Hermodsson, K. Hellstrand

**Affiliations:** Department of Surgery, Sahlgrenska Hospital, Gothenburg, Sweden.

## Abstract

Eleven patients with disseminated midgut carcinoid tumour disease were subjected to hepatic artery embolisation. In six patients, lymphocytosis with a predominance of NK cells occurred and the cytotoxic activity of isolated lymphocytes increased. A relation between NK cell accumulation and subsequent radiological and biochemical response was observed, and it is suggested that anti-tumour mechanisms other than ischaemia may contribute to the therapeutic response in these patients.


© 1995 Stockton Press All rights reserved 0007–0920/95 $9.00

SHORT COMMUNICATION

### Accumulation of natural killer cells after hepatic artery embolisation in the midgut carcinoid syndrome

B Wängberg[1], H Ahlman[1], U Tylén[2], O Nilsson[3], S Hermodsson[4] and K Hellstrand[4]

*Departments of [1]Surgery, [2]Radiology, [3]Pathology and [4]Clinical Virology, Sahlgrenska Hospital, Gothenburg, Sweden.*

**Summary** Eleven patients with disseminated midgut carcinoid tumour disease were subjected to hepatic artery embolisation. In six patients, lymphocytosis with a predominance of NK cells occurred and the cytotoxic activity of isolated lymphocytes increased. A relation between NK cell accumulation and subsequent radiological and biochemical response was observed, and it is suggested that anti-tumour mechanisms other than ischaemia may contribute to the therapeutic response in these patients.

**Keywords:** NK cells; carcinoid tumours; hepatic artery embolisation

Hepatic artery embolisation is a well-documented method in the treatment of patients with disseminated midgut carcinoid tumours. After embolisation, an increased urinary excretion of 5-hydroxyindoleacetic acid (5-HIAA) indicates tumour cell necrosis with subsequent release of serotonin (5-HT) (Ahlman *et al.*, 1991). In previous studies, embolisation therapy has been instituted to obtain symptom relief at a late stage of the disease and thus added little to survival (Coupe *et al.*, 1989). At our unit all patients with bilobar hepatic metastases during the last 6 years have undergone embolisation subsequent to primary surgery, with octreotide as adjuvant therapy (200 μg s.c. daily). In a follow-up study of the therapeutic response it was shown that the patients could be divided into two groups: (I) responders with more than 50% tumour reduction, evaluated by computerised tomography (CT) 3 months post embolisation, and a pronounced reduction in 5-HIAA excretion (80 ± 3%); (II) non-responders with less than 50% tumour reduction, or progressive disease, and a moderate 5-HIAA reduction (28 ± 12%). The first group of patients had a therapeutic response of much longer duration (Wängberg *et al.*, 1993). The therapeutic effect has been attributed to ischaemic damage to tumour cells. However, in three patients we observed ***bilateral*** tumour regression after ***unilateral*** embolisation, which focused our interest on potential activation of systemic anti-tumour mechanisms.

Natural killer (NK) cells are a subset of lymphocytes that kill tumour cells in a non-MHC-restricted fashion (Trinchieri, 1989). NK cells are non-T ($CD3^-$) lymphocytes that express CD56, a surface glycoprotein which represents an isoform of the neural cell adhesion protein (N-CAM), and CD16, a low-affinity cell-surface receptor for the Fc part of IgG. Functions of NK cells are positively regulated by 5-HT, the main product of midgut carcinoid tumour cells. 5-HT effectively augments the cytotoxic, proliferative and lymphokine-producing activities of NK cells by reversing a cell contact-dependent, suppressive signal from phagocytes (Hellstrand and Hermodsson, 1987, 1993). Against this background, we chose to monitor the frequency of lymphocytes with NK cell phenotype ($CD3^-/56^+$) and NK cell cytotoxicity against susceptible tumour target cells in the peripheral venous blood of patients with hepatic carcinoid metastases subjected to hepatic artery embolisation.

Correspondence: B Wängberg
Received 6 June 1994; revised 12 September 1994; accepted 1 November 1994

### Materials and methods

Eleven patients with hepatic metastases of midgut carcinoid tumours and markedly elevated urinary 5-HIAA excretion (759 ± 195 μmol 24 $h^{-1}$, reference value <50 μmol 24 $h^{-1}$) were treated with hepatic artery embolisation. Since the patients served as their own controls, venous blood was drawn from a central venous catheter immediately *before* and 15 min *after* unilateral embolisation. Mononuclear cells (MNCs) were isolated using Ficoll–Hypaque density gradient centrifugation (Hellstrand and Hermodsson, 1993). The NK cell-mediated cytotoxicity of MNC was analysed using K562 erythroleukaemic target cells as described in detail previously (Hellstrand and Hermodsson, 1987). Three MNC to target cell (E/T) ratios were used and results plotted. The percentage of killed tumour cells (per cent cell lysis) was calculated at an E/T ratio of 20:1. The frequency of lymphocytes with T- and NK-cell phenotype was analysed by use of flow cytometry (FACSort, Becton Dickinson, Stockholm, Sweden) after staining of lymphocyte or whole-blood specimens with fluorescein isothiocyanate (FITC)-conjugated anti-CD3 and PE-conjugated anti-CD56. In some patients the frequency of $CD56^+$ NK cells carrying the CD16 antigen was analysed using FITC-conjugated anti-CD16. All patient samples were analysed using fresh cells immediately after each embolisation and without knowledge of the clinical response status. Clinical responses were evaluated biochemically and radiologically (CT) at 3 months post embolisation. Patients with more than 50% tumour reduction, as evaluated by CT, *and* more than 50% reduction of the urinary 5-HIAA excretion (μmol 24 $h^{-1}$) were classified as responders.

### Results

In 6 of the 11 patients (54%) a marked lymphocytosis occurred within 15 min after the onset of embolisation. Analyses of the frequency of T cells ($CD3^+/56^-$), NK cells ($CD3^-/56^+$), null cells ($CD3^-/56^-$) and non-MHC-restricted T cells ($CD3^+/56^+$) by use of flow cytometry revealed that the predominant accumulating cell type was NK cells. The vast majority of these cells (85%) also carried the CD16 antigen (Figure 1). Accumulating NK cells were almost exclusively of the $CD56^{dim}$ phenotype. In most patients the proportion of $CD3^+/56^+$ cells ('non-MHC-restricted T cells') was less than 5% of gated lymphocytes and did not change after embolisation. The cytotoxic activity of isolated MNCs against NK cell-susceptible target cells was augmented in parallel with the increased number of NK cells (Figure 2). The cytotoxicity in

**Figure 1** Effect of hepatic artery embolisation on NK-cell markers in peripheral blood lymphocytes. Cells from whole-blood specimens were obtained from peripheral venous blood before (left) and after embolisation (right). The cells were stained with FITC-conjugated anti-CD16 and PE-conjugated CD56 (upper two maps) or FITC-conjugated anti-CD3 and PE-conjugated anti-CD56 (lower two maps) and analysed by flow cytometry. Data show the proportion of gated lymphocytes with each phenotype in a representative patient with tumour regression. The *x*- and *y*-axes are 4-decade log scales. This patient had an increase of NK cells from 18.0 to 48.0% and in NK-cell cytotoxicity from 22.1 to 60.5 (per cent lysis). Urinary excretion of 5-HIAA decreased by 75% and the tumour reduction on CT exceeded 50%.

**Figure 2** Effects of hepatic artery embolisation on NK cells. The left panel shows the frequency (%) of NK cells (with $CD3^-/56^+$ phenotype) in peripheral blood lymphocytes before and after embolisation (cf. Figure 1). The right panel shows NK-cell cytotoxicity of mononuclear cells from individual patients against K562 target cells, expressed as the percentage of killed tumour cells at an MNC to target cell ratio of 20:1. Filled dots represent patients with objective clinical response, evaluated biochemically and radiologically at 3 months post embolisation.

relation to number of NK cells was not changed after embolisation (data not shown), indicating that the NK cells recruited after embolisation were functionally similar to the pre-existing population in peripheral blood.

When evaluated biochemically and radiologically 3 months post embolisation, six patients were classified as responders and five as non-responders. The accumulation of NK cells and the clinical response seemed to be related, except in one patient with rapidly progressive extrahepatic disease (Figure 2). Interestingly, in contrast to all other patients, $CD3^+/56^+$ cells accumulated (9.0–25.8%) in this patient in parallel with the accumulation of NK cells (14.5–31.3%). The mean NK cell cytotoxicity (per cent cell lysis) before and after embolisation among the *responders* was 17.7% and 40.3% respectively. The mean frequency of lymphocytes with NK cell phenotype increased from 13.6% to 32.2%. Among the *non-responders* the NK cell cytotoxicity, before and after embolisation, was 15.0% and 20.8% and the corresponding frequency of NK cells was 12.3 and 16.8% respectively.

### Discussion

Previous studies have shown a decreased number and a reduced cytotoxicity of NK cells after surgery with a second reduction in NK-cell activity after subsequent chemotherapy (Lukomska *et al.*, 1983; Brenner and Margolese, 1991; Pollock *et al.*, 1991). The present study demonstrates a remarkable accumulation of NK cells in peripheral blood after hepatic artery embolisation and suggests that immunological anti-tumour mechanisms may be initiated by embolisation in certain patients with the midgut carcinoid syndrome. An immunological response rate of 54% is in accordance with the findings at earlier clinical follow-up studies in which half of the patients had a pronounced tumour reduction of long duration. The immunological response may be amplified by the release of tumour cell products, e.g. 5-HT, interleukin 2 (IL-2) and tachykinins. In two patients studied, the number of NK cells remained elevated when analysed 3-10 days post embolisation. It cannot be excluded that two unrelated phenomena are observed, i.e. one group of patients has an anti-tumour response and in these patients NK cells are mobilised perhaps from stores within the liver. However, the clinical follow-up indicates that the immunological response may serve as an early marker of the therapeutic effect. The mere presence of the demonstrated NK-cell accumulation may contradict the immediate use of chemotherapy following embolisation or a combined treatment strategy, e.g. chemoembolisation.

**Acknowledgements**

Supported by the Swedish MRC (5220), the Swedish Cancer Society (2998), the Swedish Medical Society, the Landmann Donation, the Assar Gabrielsson Foundation and the Sahlgrenska Hospital Research Foundations.

### References

AHLMAN H, WÄNGBERG B, JANSSON S, STENQVIST O, GETERUD K, TYLÉN U, CAIDAHL K, SCHERSTÉN T AND TISELL LE. (1991). Management of disseminated midgut carcinoid tumours. *Digestion*, **49**, 78–96.

BRENNER BG AND MARGOLESE RG. (1991). The relationship of chemotherapeutic and endocrine intervention on natural killer cell activity in human breast cancer. *Cancer*, **68**, 482–488.

COUPE M, HODGSON H, HEMINGWAY A AND ALLISON D. (1989). The effect of hepatic artery embolisation on survival in the carcinoid syndrome. *J. Intervent. Radiol.*, **4**, 179–181.

HELLSTRAND K AND HERMODSSON S. (1987). Role of serotonin in the regulation of human natural killer cell cytotoxicity. *J. Immunol.*, **139**, 869–875.

HELLSTRAND K AND HERMODSSON S. (1993). Serotonergic 5-$HT_{1A}$-receptors regulate a cell-contact-mediated interaction between natural killer cells and monocytes. *Scand. J. Immunol.*, **37**, 7–18.

LUKOMSKA B, OLSZEWSKI WL, ENGESET A, KOLSTAD P. (1983). The effect of surgery and chemotherapy on blood NK cell activity in patients with ovarian cancer. *Cancer*, **51**, 465–469.

POLLOCK RE, LOTZOVA E AND STANFORD SD. (1991). Mechanism of surgical stress impairment of human perioperative natural killer cell cytotoxicity. *Arch. Surg.*, **126**, 338–342.

TRINCHIERI G. (1989). Biology of natural killer cells. *Adv. Immunol.*, **47**, 187–376.

WÄNGBERG B, GETERUD K, NILSSON O, JANSSON S, DAHLSTRÖM A, TYLÉN U AND AHLMAN H. (1993). Embolisation therapy in the midgut carcinoid syndrome: just tumour ischaemia? *Acta Oncol.*, **32**, 251–256.


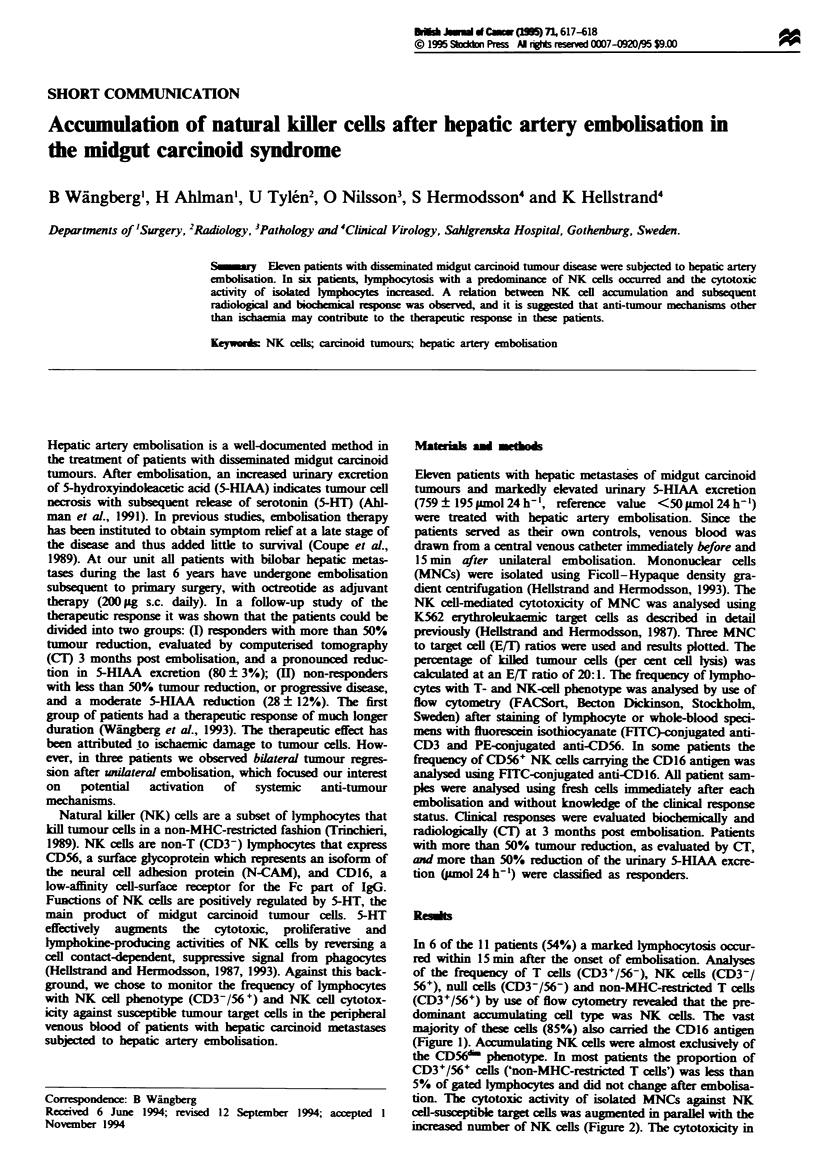

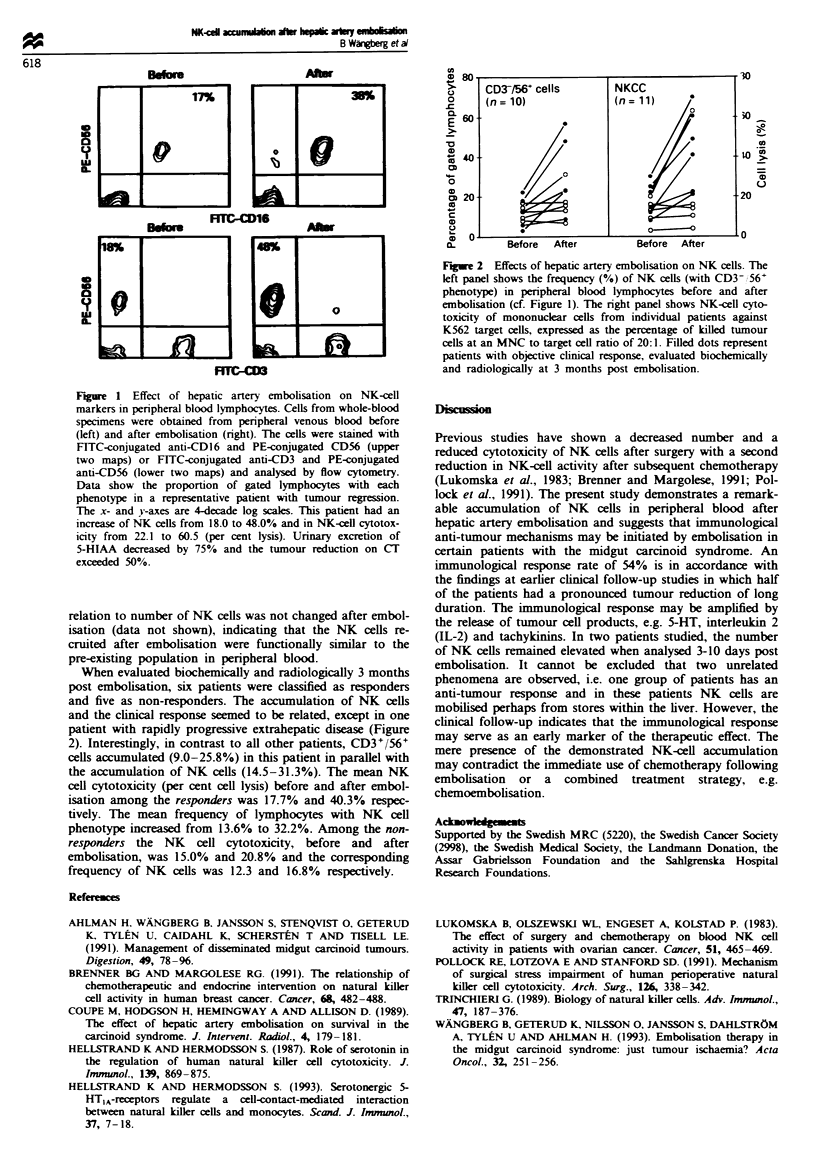


## References

[OCR_00236] Ahlman H., Wängberg B., Jansson S., Stenqvist O., Geterud K., Tylén U., Caidahl K., Scherstén T., Tisell L. E. (1991). Management of disseminated midgut carcinoid tumours.. Digestion.

[OCR_00244] Brenner B. G., Margolese R. G. (1991). The relationship of chemotherapeutic and endocrine intervention on natural killer cell activity in human breast cancer.. Cancer.

[OCR_00252] Hellstrand K., Hermodsson S. (1987). Role of serotonin in the regulation of human natural killer cell cytotoxicity.. J Immunol.

[OCR_00257] Hellstrand K., Hermodsson S. (1993). Serotonergic 5-HT1A receptors regulate a cell contact-mediated interaction between natural killer cells and monocytes.. Scand J Immunol.

[OCR_00263] Lukomska B., Olszewski W. L., Engeset A., Kolstad P. (1983). The effect of surgery and chemotherapy on blood NK cell activity in patients with ovarian cancer.. Cancer.

[OCR_00268] Pollock R. E., Lotzová E., Stanford S. D. (1991). Mechanism of surgical stress impairment of human perioperative natural killer cell cytotoxicity.. Arch Surg.

[OCR_00273] Trinchieri G. (1989). Biology of natural killer cells.. Adv Immunol.

[OCR_00277] Wängberg B., Geterud K., Nilsson O., Jansson S., Dahlström A., Tylén U., Ahlman H. (1993). Embolisation therapy in the midgut carcinoid syndrome: just tumour ischaemia?. Acta Oncol.

